# Metabolic Syndrome, Alcohol Consumption and Genetic Factors Are Associated with Serum Uric Acid Concentration

**DOI:** 10.1371/journal.pone.0097646

**Published:** 2014-05-14

**Authors:** Blanka Stibůrková, Markéta Pavlíková, Jitka Sokolová, Viktor Kožich

**Affiliations:** Institute of Inherited Metabolic Disorders, First Faculty of Medicine, Charles University in Prague and General University Hospital in Prague, Prague, Czech Republic; University of Maryland School of Medicine, United States of America

## Abstract

**Objective:**

Uric acid is the end product of purine metabolism in humans, and increased serum uric acid concentrations lead to gout. The objective of the current study was to identify factors that are independently associated with serum uric acid concentrations in a cohort of Czech control individuals.

**Methods:**

The cohort consisted of 589 healthy subjects aged 18–65 years. We studied the associations between the serum uric acid concentration and the following: (i) demographic, anthropometric and other variables previously reported to be associated with serum uric acid concentrations; (ii) the presence of metabolic syndrome and the levels of metabolic syndrome components; and (iii) selected genetic variants of the *MTHFR* (c.665C>T, c.1286A>C), *SLC2A9* (c.844G>A, c.881G>A) and *ABCG2* genes (c.421C>A). A backward model selection procedure was used to build two multiple linear regression models; in the second model, the number of metabolic syndrome criteria that were met replaced the metabolic syndrome-related variables.

**Results:**

The models had coefficients of determination of 0.59 and 0.53. The serum uric acid concentration strongly correlated with conventional determinants including male sex, and with metabolic syndrome-related variables. In the simplified second model, the serum uric acid concentration positively correlated with the number of metabolic syndrome criteria that were met, and this model retained the explanatory power of the first model. Moderate wine drinking did not increase serum uric acid concentrations, and the urate transporter *ABCG2*, unlike *MTHFR*, was a genetic determinant of serum uric acid concentrations.

**Conclusion:**

Metabolic syndrome, moderate wine drinking and the c.421C>A variant in the *ABCG* gene are independently associated with the serum uric acid concentration. Our model indicates that uric acid should be clinically monitored in persons with metabolic syndrome.

## Introduction

An increased serum uric acid (UA) concentration (>416 µmol/L in men and >340 µmol/L in children and women) is an important risk factor for gout and has other significant associations with human disorders, such as cardiovascular and renal diseases [1], [2]. Links between increased serum UA concentrations and the levels of the individual components of metabolic syndrome (MS) have been reported [3], [4]. Serum UA concentrations have also been linked to many ageing-related illnesses and to brain function, although the direction of the association is unclear. It has been suggested that higher UA concentrations may be associated with increased performance on memory-related tasks [5]. Studies have provided evidence that UA and gout lower the risk of developing Parkinson's disease [6], [7]. Many factors, including obesity, body mass index and alcohol consumption, are known to influence serum UA concentrations [4], [8]–[11]. In addition, data from numerous studies suggest that serum UA concentrations are markedly heritable; the proportion of variance explained by a shared genetic background ranges from 0.38–0.63 [12]–[15].

UA is the end product of purine metabolism in humans and higher primates; in contrast, other mammals further metabolize urate to allantoin. The absence of the hepatic enzyme uricase (urate oxidase) and efficient renal UA reabsorption contributes to the ten-fold higher UA blood levels in humans compared to those of other mammals. UA is a powerful scavenger of peroxyl radicals, hydroxyl radicals and singlet oxygen in human biological fluids. The balance between UA production and excretion determines serum UA concentrations; the daily production of urate is approximately 1000 mg (6 mmol) in adults. Approximately 75% of UA excretion occurs via the kidney, and the gastrointestinal tract eliminates 25%. The renal excretion of UA depends on the balance between filtration, secretion and reabsorption. Transporters for urate localize in the proximal tubule, where specific transporter proteins in the apical and basolateral membranes secrete and extensively reabsorb urate. As a result, approximately 10% of the UA filtered through the glomerular membranes is excreted.

Many studies have shown an association between alcohol consumption and hyperuricemia, which is a common precipitating cause of acute gouty arthritis [16], [17]. Alcohol consumption is believed to elevate serum UA through two mechanisms: the accelerated synthesis of UA from adenosine, which is produced after the degradation of adenosine triphosphate to adenosine monophosphate, and reduced urinary excretion due to the elevation of blood lactate caused by the oxidation of ethanol [18].

Over the past decade, genome-wide association studies and meta-analyses have led to a massive increase in our knowledge of the common genetic variants that influence serum UA concentrations. However, detailed knowledge of the degree to which genetic variants predict serum UA concentrations and of the functional pathway through which these variants may alter serum UA remains limited. The genes that influence the level of serum UA via renal UA excretion are those for the urate transporters, primarily those encoded by the *SLC2A9*, *SLC22A12* and *ABCG2* genes. The URAT1 transporter, the product of *SLC22A12*, was identified in 2002 as the crucial transporter involved in UA reabsorption. The URAT1 transporter regulates blood UA levels and plays a central role in the reabsorption of UA from the glomerular filtrate [19]. Loss-of-function mutations in the *SLC22A12* gene cause renal hypouricemia type 1, OMIM #220150 [20], [21]. In 2008, the primary role of *SLC2A9* in UA reabsorption was identified [22], [23]. Recent results revealed significant correlations between genetic variants in *SLC2A9* and serum UA levels, the excreted fraction of UA, blood pressure, body mass index and gout [22]–[24]. The *SLC2A9* genetic variants are responsible for a portion of the variance in serum UA concentrations: 5–6% in females and 1–2% in males [22]–[24]. Mutations in the *SLC2A9* gene cause renal hypouricemia type 2, OMIM #612076 [25]–[27]. UA secretion is mostly determined by the transporter ATP-binding cassette subfamily G member 2 (ABCG2/BCRP). Variant c.421C>A (rs2231142, p.Q141K) results in a reduction of the urate transport rate by 53% compared with that for the wild-type *ABCG2* and causes approximately 10% of the hyperuricemia and gout cases in Caucasians [28].

Although most genes associated with UA are involved in the renal urate transport system, an association between homozygosity for the common genetic variant c.665C>T (known as c.677C>T; rs1801133, p.A222V) of methylenetetrahydrofolate reductase (*MTHFR*, 1p36.22, EC 1.5.1.20) and the serum UA concentration was also reported [29]–[32]. MTHFR is a cytoplasmic enzyme that catalyzes the reduction of 5,10-methylenetetrahydrofolate to 5-methyltetrahydrofolate, the primary methyl donor for the methylation of homocysteine to methionine. It has been speculated that *MTHFR* allelic variants may be confounding genetic factors in the relationship between serum UA and hypertension [33].

Even with extensive research *via* different approaches into the basis of serum UA concentrations, a complete understanding of serum UA production, excretion and interference has not yet been reached. Our study was aimed at answering the following unresolved questions: (*a*) What is the relationship between serum UA and demographic, anthropometric and other variables? (*b*) What is the relationship between serum UA and metabolic syndrome? (c) What is the influence of genetic determinants on serum UA? In our study, we examined the associations between serum UA concentrations and a wide range of variables in a cohort from central Europe. We modeled the associations between UA and (i) demographic, anthropometric and other variables previously reported to be associated with serum UA (referred to as conventional variables); (ii) the presence of metabolic syndrome (MS) and the levels of MS components and (iii) selected genetic variants in the *SLC2A9*, *ABCG2* and *MTHFR* genes. We have adopted a complex approach, combining all these variables into one regression model.

## Materials and Methods

### Subjects

We have used the data and genomic DNA samples from subjects of a previously described study [34]. All participants were 18–65 years old, residents of Central Bohemia or Prague with no history and signs of coronary artery disease, peripheral arterial disease and stroke. They were recruited from health clinics, general practitioner's offices, on community health days and by advertising in company bulletins. This study collected various anamnestic, clinical and biochemical parameters and questionnaire-based data about lifestyle, e.g., smoking, the consumption of alcoholic beverages, the consumption of fruit and vegetables, caloric restriction and vitamin use. The original cohort consisted of 591 subjects. Two subjects did not have recorded UA concentrations and were excluded from the analysis, resulting in a total of 589 subjects. Their characteristics are summarized in Table 1. The median UA concentrations were 330 µmol/L in men (n = 285) and 232 µmol/L in women (n = 304). The UA concentrations of 536 subjects fell within the normal UA concentration range. There were 3 subjects (all women) with UA concentrations below the reference range and 50 subjects (35 men and 15 women) with UA concentrations above the reference range and all remained in the study. The study was approved by the Ethics Committee of Charles University – First Faculty of Medicine; all subjects gave their informed written consent.

**Table 1:** Overview of the UA concentrations and conventional and MS related variables in the study sample.

	Median (1^st^, 3^rd^ quartile) or N (%)		
	All	Men	Women
**Uric acid concentration**, *µmol/L*	272 (223, 337)	330 (279, 380)	232 (197, 267)
**Conventional predictors**			
Sex	-	285 (48%)	304 (52%)
Climax (women only), *yes/no*	-	-	145 (48%)
Age, *year*	50 (43, 55)	50 (43, 55)	49 (42, 55)
Alcohol consumption – beer, *0.5 L drink/week* **	1 (0, 4)	4 (1, 8)	0 (0, 1)
- consumers only, *0.5 L drink/week* **	3 (2, 7)	5 (2, 10)	2 (1, 3)
Alcohol consumption – wine, *0.2 L drink/week* **	0 (0, 1)	0 (0, 1)	0 (0, 1)
- consumers only, *0.2 L drink/week* **	2 (1, 3)	2 (1, 4)	1.5 (1, 2.5)
Smoking – smoker; *yes/no*	115 (20%)	59 (21%)	56 (18%)
Smoking – former smoker; *yes/no*	133 (23%)	83 (29%)	50 (16%)
Smoking inpackyears; *pack·year*	0 (0, 3287)	0 (0, 4657)	0 (0, 1046)
- current and former smokers only; *pack·year*	4200 (1621, 7739)	4703 (2214, 10958)	3653 (776, 5798)
Allopurinol users, *yes/no*	7 (1%)	6 (2%)	1 (0.3%)
Diuretics users, *yes/no*	29 (5%)	10 (4%)	19 (6%)
Acetylsalicylic acid users, *yes/no*	17 (3%)	7 (2%)	10 (3%)
HA users (women only), *yes/no*	-	-	36 (12%)
SHT users (women only), *yes/no*	-	-	74 (24%)
Hormones (women only), *yes/no*	-	-	110 (36%)
Serum creatinine, *µmol/L*	83 (74, 92)	90 (82, 97)	76 (71, 85)
**MS related predictors**			
BMI, *kg/m^2^*	25.9 (23.4, 28.4)	26.4 (24.5, 28.4)	25.2 (22.3, 28.0)
WHR, *m/m*	0.85 (0.80, 0.90)	0.90 (0.86, 0.94)	0.80 (0.77, 0.84)
Obesity*, *yes/no*	389 (66%)	215 (75%)	174 (57%)
Systolic pressure, *mm Hg*	125 (120, 140)	125 (120, 140)	120 (115, 135)
Diastolic pressure, *mm Hg*	80 (75, 90)	80 (80, 90)	80 (70, 85)
Hypertension, *yes/no*	79 (13%)	41 (14%)	38 (13%)
Hypertension treatment, *yes/no*	59 (10%)	30 (11%)	29 (10%)
Hypertension*, *yes/no*	312 (53%)	163 (57%)	149 (49%)
Glycemia, *mmol/L*	5.04 (4.69, 5.44)	5.21 (4.84, 5.62)	4.90 (4.60, 5.31)
Diabetes mellitus, *yes/no*	23 (4%)	15 (5%)	8 (3%)
DM treatment, *yes/no*	9 (2%)	7 (2%)	2 (1%)
Hyperglycemia*, *yes/no*	115 (19%)	77 (27%)	38 (13%)
Total cholesterol, *mmol/L*	5.36 (4.79, 6.07)	5.38 (4.80, 6.20)	5.33 (4.76, 5.90)
HDL cholesterol, *mmol/L*	1.45 (1.24, 1.72)	1.32 (1.18, 1.52)	1.59 (1.35, 1.87)
LDL cholesterol, *mmol/L*	3.23 (2.67, 3.84)	3.34 (2.71, 3.92)	3.09 (2.60, 3.79)
Reduced HDL-cholesterol*, *yes/no*	86 (15%)	27 (9%)	59 (19%)
Triacylglycerols, *mmol/L*	1.21 (0.91, 1.80)	1.44 (1.02, 2.06)	1.10 (0.81, 1.48)
Hypertriacylglycerolemia*, *yes/no*	170 (29%)	112 (39%)	58 (19%)
Hyperlipidemia, *yes/no*	144 (24%)	82 (29%)	62 (20%)
GGT, *µkat/L*	0.53 (0.42, 0.72)	0.63 (0.48, 0.89)	0.45 (0.38, 0.62)
Number of MS criteria			
- 0 criteria, *yes/no*	116 (20%)	38 (13%)	78 (26%)
- 1 criteria, *yes/no*	135 (23%)	54 (19%)	81 (27%)
- 2 criteria, *yes/no*	160 (27%)	86 (30%)	74 (24%)
- 3 criteria, *yes/no*	107 (18%)	67 (24%)	40 (13%)
- 4 criteria, *yes/no*	52 (9%)	30 (10%)	22 (7%)
- 5 criteria, *yes/no*	17 (3%)	9 (3%)	8 (3%)
MS, *yes/no*	176 (30%)	103 (36%)	70 (23%)

*Criteria of MS: 1. Hypertension, systolic presure ≥130 mmHg and/or diastolic presure ≥85 mmHg and/or treatment of hypertension; 2. Reduced HDL-cholesterol, HDL cholesterol<1.0 (<1.30, resp.) mmol/L for men (women, resp.); 3. Hypertriacylglycerolemia, triacylglycerol ≥1.7 mmol/L; 4. Hyperglycemia, glycemia ≥5.6 mmol/L and/or treated diabetes mellitus; 5. Obesity, BMI ≥25 kg/m2 and/or WHR ≥0.90 (≥0.85) for men (or women, respectively).

**Throughout the analysis and interpretation of the effect of alcohol consumption we used the number of drinks per day,however, for clarity of presentation, the alcohol consumption is shown in number of drinks per week.

### Clinical and biochemical investigations

UA in the serum was measured using a uricase enzymatic method by an auto-analyzer. We studied the associations between the serum UA concentration and the following demographic characteristics, metabolite levels, conventional risk factors and genetic risk factors: (i) conventional variables—sex, age, alcohol consumption (beer and wine), smoking status and number of packyears, allopurinol use, diuretic use, acetylsalicylic acid consumption, hormonal treatment, hormonal contraception and serum creatinine levels, (ii) metabolic syndrome-related variables—BMI, WHR (waist-to-hip ratio), systolic and diastolic pressure, hypertension, treatment of hypertension, hyperglycemia, diabetes mellitus and its treatment, total cholesterol, HDL and LDL cholesterol, triacylglycerol levels, hyperlipidemia and the gamma-glutamyltransferase (GGT) concentration and (iii) selected genetic variants (see above). For the second analysis, we constructed a new variable called metabolic syndrome (the number of metabolic criteria met) and used it instead of the MS-related variables. We used the following common criteria for MS reported by several organizations, including the WHO, the IDF and the AHA/NHLBI [35]: abdominal obesity, elevated triglycerides (≥1.7 mmol/L), reduced HDL-cholesterol (<1.00 mmol/L in males and <1.3 mmol/L in females), elevated blood pressure (systolic pressure ≥130, diastolic pressure ≥85 mmHg or treatment for hypertension) and elevated fasting glucose concentration (≥5.6 mmol/L and/or treated for diabetes mellitus). Because waist circumference data were not available, we instead used the criteria of a BMI ≥25 kg/m^2^ and/or a WHR ≥0.90 for men and ≥0.85 for women [36] as indicators of obesity.

### Genotype analysis

The frequent genetic variants of genes encoding the urate transporter *SLC2A9* (c.844G>A (rs16890979, p.V282I) and c.881G>A (rs3733591, p.R294H)), the urate transporter *ABCG2* (c.421C>A (rs2231142, p.Q141K)) and the *MTHFR* enzyme (c.665C>T (known as c.677C>T; rs1801133, p.A222V) and c.1286A>C (known as c.1298A>C; rs1801131, p.E429A)) were selected for genotype analysis. The selected genetic variants of the urate transporters were analyzed by PCR with allele-specific primers (ARMS-PCR); the primers and conditions are given in Table S1. The *MTHFR* variants were determined in a previous study [34].

### Statistical analyses

In total, 30 potential predictors were included in the analysis. For statistical purposes, the UA concentration and the glucose, GGT and triglyceride levels were logarithmically transformed. The association between the variables and the serum UA concentration was evaluated in two steps. First, single-predictor linear regression models were utilized with the UA concentration as the response variable and one predictor variable adjusted for sex (including possible interactions with sex). Then, the backward model selection procedure was used to build a multiple linear regression model. This model was based on the set of variables that showed association (p-value <0.1) in the single-predictor models, together with all interaction terms with sex. In the second multiple linear regression analysis, we started with the same large set of variables in interaction with sex, but we replaced the predictor variables related to metabolic syndrome with the number of MS criteria met and repeated the backward selection procedure.

We assessed the relative effect of the genetic component in the final regression models by analyzing the proportion of the explained variance relative to the adjusted R^2^ value. The logarithmic transformation of the UA concentration made it difficult to interpret the values of the coefficients in the multiple regression models. We therefore calculated the model-predicted mean change in UA concentration when the studied variable increased by one unit while the values of the other variables remained the same for an individual whose original UA concentration was 326 µmol/L (the mean UA concentration in men; the UA concentration in women was calculated from the model).

The level of statistical significance was set to 0.05 for the coefficients in the multiple regression analysis models. All analyses were performed in the statistical language and environment R, version 2.14.0 (http://www.R-project.org).

## Results

### Single-predictor regression analysis

Table S2 shows the association of individual variables with the UA concentration in the single-predictor regression analysis (with sex as the covariate). In our model the majority of conventional and MS-related variables were associated with the UA concentration indicating a high reliability of data.

Genetic factors were significantly associated with the serum UA concentrations. The allelic and genotype frequencies and geometric means of serum UA concentrations in wild-type homozygotes, heterozygotes and variant homozygotes for different loci are shown in Table 2. All genotypes were in Hardy-Weinberg equilibrium with the exception of the *MTHFR* variant c.665C>T in males (p<0.022); since the genotyping errors are unlikely this observation may be due to the population stratification or by random occurrence. There was a significant additive effect of *ABCG2* c.421C>A (p-value <0.001) on the increase in the UA concentration. The single-predictor analysis of the effect of the *SLC2A9* c.881G>A variant showed a possible additive effect on the decrease in the serum UA concentration (p-value <0.001), whereas the *SLC2A9* c.844G>A variant did not show any effect on the UA concentration. No significant relationship was found between the *MTHFR* variant c.665C>T and the UA concentration. Homozygous individuals with the *MTHFR* c.1286A>C variant had significantly lower UA concentrations (p-value  = 0.025) than those of the remainder of the study population. However, when *MTHFR* c.1286A>C was considered as a three-level factor (A/A, A/C and C/C genotypes), the model p-value was greater than the significance level (p-value  = 0.092).

**Table 2:** Allelic and genotype frequencies and geometric means of serum UA concentrations in wild-type homozygotes (WW), heterozygotes (WM) and variant homozygotes (MM) for different genotypes by sex.

		Number of individuals			Mutant allele frequency	Hardy-Weinberg equilibrium	Geometric means of serum UA concentration, µmol/L		
		WW	WM	MM		p-value	WW	WM	MM
Men	*MTHFR* c.665C>T	106	150	29	36.5%	0.022	323	326	333
	*MTHFR* c.1286A>C	127	135	23	31.8%	0.117	332	322	321
	*ABCG2* c.421C>A	230	52	3	10.2%	0.975	320	346	489
	*SLC2A9* c.881G>A	180	87	18	21.6%	0.098	331	320	301
	*SLC2A9* c.844G>A	170	100	15	22.8%	0.953	339	308	301
Women	*MTHFR* c.665C>T	137	137	30	32.4%	0.616	226	228	251
	*MTHFR* c.1286A>C	137	131	36	33.4%	0.586	230	235	208
	*ABCG2* c.421C>A	245	56	3	10.2%	0.920	226	241	257
	*SLC2A9* c.881G>A	188	102	14	21.4%	0.972	229	228	239
	*SLC2A9* c.844G>A	187	106	11	21.1%	0.393	237	219	203

**Figure 1:**
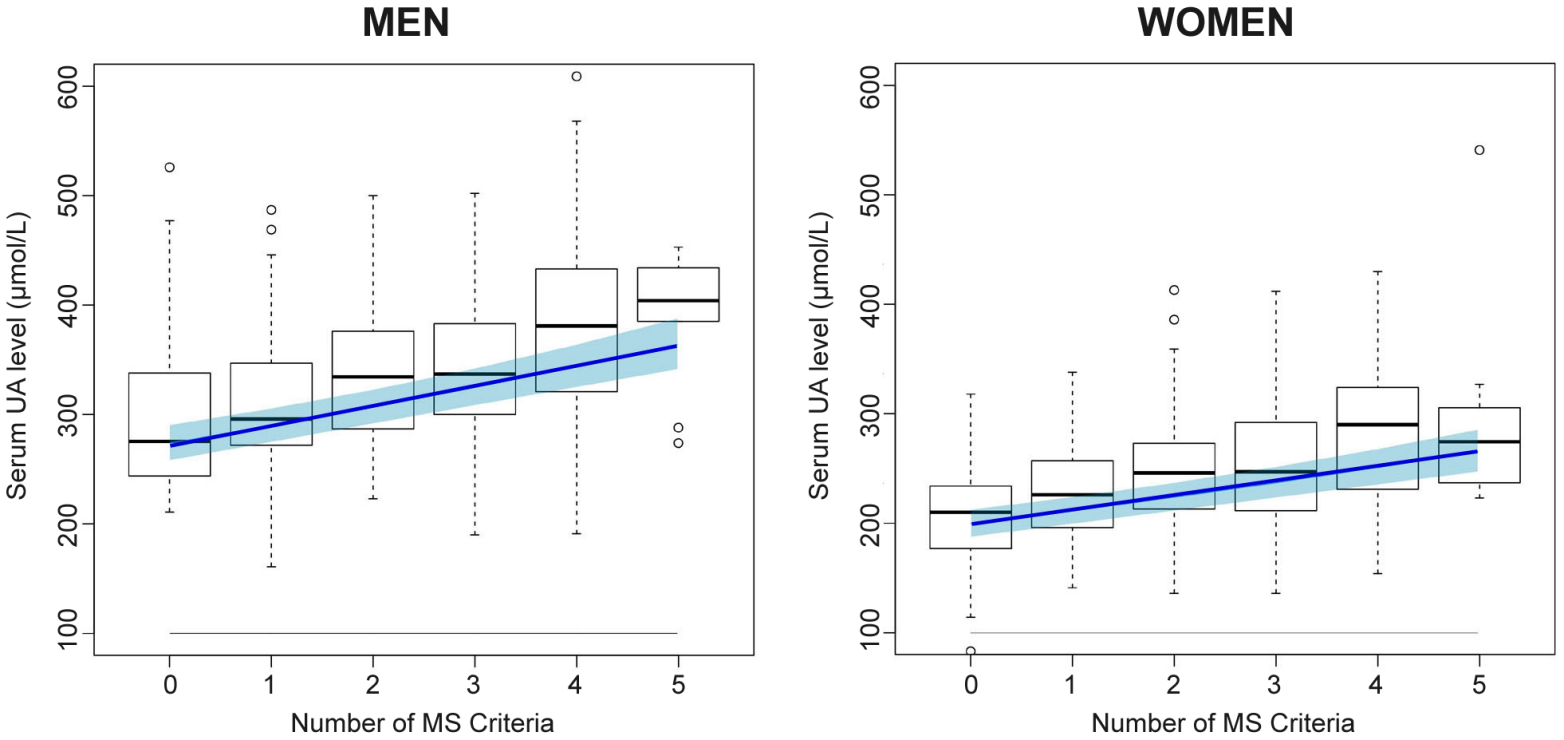
Boxplot: estimate and confidence limits – for men and women, respectively. The boxes show median (black bar), quartiles (box limits) and extreme values of uric acid concentrations for individuals with 0, 1, 2, 3, 4 or 5 metabolic syndrome criteria met. The line shows estimated geometric mean of the uric acid concentrations, as calculated from Model 2. The band shows 95% confidence limit for the estimate.

In the study population, 30% of the subjects had MS (106 men and 70 women met three or more MS criteria), and 70% of the subjects did not have MS (178 men and 233 women). The box and whisker plot in Figure 1 shows the distribution of the serum UA concentrations based on the number of MS criteria met by the study subjects. Although the figure is not adjusted for other potentially influential factors, it suggests the tendency for the serum UA concentration to increase as more MS criteria were met.

### Multiple regression analysis

To assess the independent contributions of the variables to the UA concentration, a multiple linear regression model was built (Model 1 in Table 3 and Table S2). The variability in the serum UA concentrations was best explained by sex; age; BMI; hypertension; hyperglycemia; the serum levels of triacylglycerols, creatinine and GGT; allopurinol intake, *MTHFR* c.1286A>C, *ABCG2* c.421C>A, *SLC2A9* c.844G>A and *SLC2A9* c.881G>A. The adjusted coefficient of determination (R^2^) was 0.59. The genetic variables together explained approximately 4.5% of the total variability.

**Table 3:** Estimate of the effects of different predictors on the serum UA concentration.

	Model 1		Model 2	
	Change for value, *µmol/L*		Change for value, *µmol/L*	
	326 µmol/L*	242 µmol/L**	326 µmol/L*	238 µmol/L**
	Man	Woman	Man	Woman
**Conventional predictors**				
Sex, *female vs. male*	−84***	n.a.	−88***	n.a.
Climax, *yes/no*	n.a.	24,8	n.a.	31
Age, *per 1 year increase*	−0,6	−0,4	−0,7	−0,5
Alcohol consumption				
- beer, *per 0.5 L/day increase*	-	-	11	8
- wine, *per 0.2 L/day increase*	-	-	-19	-14
Allopurinol users, *yes/no*	67	50	55	40
Diuretics users, *yes/no*	-	-	29	21
Serum creatinine, *per 10 µmol/L increase*	13	10	12	9
**MS related predictors**				
BMI, *per 1 kg/m^2^ increase*	0	4	-	-
Hypertension, *yes/no*	25	19	-	-
Glycemia, *per 1 mmol/L increase*	−7	−5	-	-
Triacylglycerols, *per 0.1 mmol/L increase*	3	2	-	-
GGT, *per 0.1 µkat/L increase*	4	3	-	-
**Number of MS criteria**				
- 1 criterion vs. 0	-	-	19	14
- 2 criteria vs. 0	-	-	39	29
- 3 criteria vs. 0	-	-	61	44
- 4 criteria vs. 0	-	-	83	61
- 5 criteria vs. 0	-	-	107	78
**Genetic predictors**				
*MTHFR* c.1286A>C				
- CC vs. (AA or AC)	−20	−15	−17	−13
*ABCG2* c.421C>A				
- CA vs. CC	39	8	27	19
- AA vs. CC	83	15	56	41
*SLC2A9* c.881G>A				
- GA vs. GG	−14	−10	−14	−10
- AA vs. GG	−28	−21	−27	−20
*SLC2A9* c.844G>A				
- GA vs. GG	−28	−21	−27	−19
- AA vs. GG	−54	−40	−51	−37

*Change of UA level per predictor unit when other explanatory variables remain the same, calculated for baseline 326 µmol/L (geometric mean of UA concentration for men in the data set)

**Change of UA level per predictor unit when other explanatory variables remain the same. The baseline for women is calculated from the Model 1 or 2, based on the baseline for men (326 µmol/L) and median BMI  = 25 for women in the data set.

***Model-predicted difference between men and women, provided all other variables remain the same. Serves for the model-predicted baseline calculation for women.

The final model contained five variables associated with MS. Four were associated with an increase in the serum UA concentration: BMI, hypertension, triacylglycerol levels and GGT levels. Hyperglycemia was associated with a slight decrease in serum UA concentration. An alternative multiple linear regression model was constructed in which all variables associated with MS were replaced by the number of MS criteria that were met, and the backwards selection model procedure was re-run. The alternative model is shown as Model 2 in Table 3 and in Table S2. The coefficient of determination was 0.53, which was very similar to Model 1. The genetic variables explained approximately 4% of the total variability in the simplified Model 2. The linear effect of MS was highly significant; this model suggests that there is an important association of MS with the serum UA concentration. Figure 1 displays the model-predicted mean values of UA and the 95% confidence intervals in men and women for the various numbers of MS criteria that were met while the other variables remained the same. In addition to MS and genetic variables, the serum UA concentration was positively associated with menopause, beer consumption (Model 2 only), the serum creatinine level, the use of allopurinol and the use of diuretics (Model 2 only), whereas it was negatively associated with female sex, age and wine consumption (Model 2 only).

In both models, the serum UA concentration was higher for the individuals with the C allele of the *ABCG2* c.421C>A gene. The increase in UA as the number of mutant alleles increased was five times higher for men than for women (Model 1; in Model 2, the interaction with sex was not statistically significant). The *SLC2A9* c.881G>A and c.844G>A variants showed negative effects on the serum UA concentrations. In both models, the serum UA concentration was lower for individuals with the *MTHFR* c.1286CC genotype.

## Discussion

In the present study, we demonstrated the following new findings: (*a*) the serum UA concentration was positively correlated with the number of MS criteria that were met, and moreover, this simplification had no substantial influence on the explanatory power of the model; (b) moderate wine drinking likely does not increase serum UA concentrations; and (c) the urate transporter *ABCG2*, unlike *MTHFR*, is one of the genetic determinants of serum UA concentrations. The evidence for an influence of two *SLC2A9* gene variants is inconclusive.

We showed that the serum UA concentration correlates positively (p<0.001) with the number of MS criteria (linear Model 2, Figure 1). Furthermore, we showed that this simplification has no substantial influence on the explanatory power of the model (the adjusted R^2^ of the simplified model is 0.53, and that of the full model is 0.59). Several studies explored the links between increased serum UA concentration and various components of MS [3]. Some studies reported that the association between UA and MS components was stronger in females than in males [4]. This result was not found in our study. Two recent studies [4], [37] explored the association between higher UA levels and future MS development in two Asian populations. The design of our study cannot resolve the direction of causality; however, our findings, together with those mentioned above, may have implications for the use of serum UA concentration as a risk marker of metabolic syndrome and *vice versa* and for screening for hyperuricemia-related problems in patients with MS in clinical practice.

Alcohol consumption in our study was analyzed separately for beer and for wine. Although the serum UA concentration increased with increasing beer intake, our results supported the previously reported solitary finding that moderate wine drinking does not increase the serum UA concentration [37]. Beer is an alcoholic beverage with high purine content, whereas wine contains a minimal amount of purines and several nonalcoholic components, including antioxidants, vasorelaxants and stimulants of anti-aggregatory mechanisms. Thus, the effect of the purines ingested from beer on the serum UA concentrations may be sufficient to augment the hyperuricemic effect of alcohol itself; however, the absence of an effect of wine may result from non-alcoholic components with antioxidant properties, such as polyphenols, which may mitigate the impact of alcohol on the serum UA concentration.

We found no significant relationship between the *MTHFR* c.665C>T variant and the UA concentration, and our finding is supported by two studies in Japanese subjects [38], [39]. However, previously reported studies demonstrated a significant association between the serum UA concentration and the *MTHFR* c.665C>T allele in other ethnic groups [29]–[32]. The relationship between *MTHFR* c.1286A>C and serum UA concentrations has not been studied. The mechanism that determines the relationship between *MTHFR* polymorphisms and serum UA concentrations is unknown. Some studies presumed that the *MTHFR* allelic variants affected *de novo* purine synthesis via 10-formyl tetrahydrofolate, with the consequent overproduction of UA by the substrate of the MTHFR reaction [32], [33]. Our finding that people with the *MTHFR* c.1286C homozygous genotype had significantly decreased UA concentrations should be considered as a topic for further exploration.

Most of the genes that influence the serum UA concentration are involved in renal excretion. Recent genome-wide association studies have identified a relationship between common genetic variants of urate transporters and either increased serum UA concentrations or primary gout. The most significant SNPs were c.421C>A in the secretion transporter *ABCG2* [41] and c.844G>A and c.881G>A in the urate reabsorption transporter *SLC2A9* [40]–[42].

Our study showed a strong association between the allelic variant c.421C>A in the *ABCG2* transporter and increasing serum UA concentrations. This effect was more pronounced in males than in females (Model 1) and was independent of all other covariates. Our results confirm that this alteration in the *ABCG2* gene is a common cause of hyperuricemia due to decreased renal urate excretion. This finding is consistent with previously reported studies in which the *ABCG2* c.421C>A variant [28], [44], [45]has been associated with hyperuricemia and gout in individuals of European, Han Chinese, Japanese and African-American ancestry [28], [40], [43], [44], however, this association was not found in Maori subjects [45]. In contrast, we found that the *SLC2A9* c.844G>A and c.881G>A variants were associated with a significant decrease in serum UA concentrations. This result is surprising, as all other studies reported increased serum UA levels or mixed results in individuals carrying c.844G>A and c.881G>A [41], [42]. More precisely, the c.844G>A variant in *SLC2A9* was associated with elevated serum uric acid concentration (especially in women) in the Framingham and Rotterdam cohorts [40], and in the island population of the Adriatic coast of Croatia [46] but not in African-Americans [40]. Variant c.881G>A in the *SLC2A9* gene was significantly associated with elevated serum uric acid concentrations and gout in the Han Chinese, Solomon Island and Japanese cohorts [42], [47], but not in the Eastern Polynesians, Western Polynesians and Europeans [41], [46]. Since the genotype frequencies in our study were similar to other studies (with the exception of the Adriatic cohort [46]) it may be hypothesized that additional factors including dietary and life-style habits, and the absence of linkage disequilibrium play role in negative association of the *SLC2A9* variants with serum UA concentrations in the Czech cohort.

Using a multiple linear regression model, we confirmed the previously reported sex-specific differences and the strong associations between serum UA concentration and age, menopause, BMI, hypertension, alcohol consumption, serum triacylglycerol levels, creatinine levels and GGT levels [4], [8]–[11], [48]–[51]. Our study also examined additional determinants of serum UA concentrations, such as MS, type of alcohol consumption and five selected genetic variants. These associations have been also studied previously, however, these variables were not analyzed simultaneously in a multiple regression model.

Our study has several strengths. First, we explored the association between serum UA concentrations and several important demographic, anamnestic, biochemical and genetic factors in a Central European (Czech) population with detailed characterization that allowed us to answer our research question and that could be generalized to the larger population. Second, we controlled for several potential confounders, such as the types of medications, that may influence serum uric acid concentrations. Third, we used a complex approach, a multiple linear regression model that provides a comprehensive estimate of the association. Different limitations of this study should also be acknowledged. First, the size of the cohort may not be sufficiently large for such detailed analysis. Second, the number of the frequent genetic variants of genes encoding the urate transporters was limited.

In conclusion, we showed that the serum UA concentration positively correlated with the number of MS criteria that were met. We found that moderate wine drinking may not increase serum UA concentrations. We confirmed that the urate transporter *ABCG2* is one of the genetic determinants of serum UA concentrations. These data may have implications for the clinical prediction of UA concentrations and may lead to personalized medical diagnoses and treatments for common diseases. An increased serum UA concentration is significantly associated with MS. In clinical practice, hyperuricemia is an indication for investigating MS criteria, and the presence of MS is an indication for investigating the serum UA concentration.

## Supporting Information

Table S1 **Genotype analysis - arrangement and primers.** The selected genotypes were determined using multiplex ARMS-PCR. The amplifications were performed in the total volume 10 µl using Combi PPP master mix (Top-Bio s.r.o., Prague, Czech Republic) supplied with allele specific primer pairs as it is shown in this table. The cycling conditions were [95/2 min;30 x (95/10 s, 68/30 s); 68/5 min] in DNA Engine Dyad PTC-220 (MJ Research, Waltham, Massachusetts). PCR products were analysed on 3% agarose gels.
(DOC)

Table S2 **The final multiple linear regression model (Model 1) and the alternative model, with MS predictors replaced by graded MS (Model 2).** * log-transformed for the model, coefficient and standard error relevant for the transformed variable. ** values for man/woman because of an interaction with gender present in the model.
(DOC)
