# Supplementary material for: Metabolic Syndrome, Alcohol Consumption and Genetic Factors Are Associated with Serum Uric Acid Concentration
Source: PLoS One. 2014 May 14;9(5):e97646. doi: 10.1371/journal.pone.0097646 (PMC4020828; doi:10.1371/journal.pone.0097646)
Supplement: Table S1 — Genotype analysis - arrangement and primers. The selected genotypes were determined using multiplex ARMS-PCR. The amplifications were performed in the total volume 10 µl using Combi PPP master mix (Top-Bio s.r.o., Prague, Czech Republic) supplied with allele specific primer pairs as it is shown in this table. The cycling conditions were [95/2 min;30 x (95/10 s, 68/30 s); 68/5 min] in DNA Engine Dyad PTC-220 (MJ Research, Waltham, Massachusetts). PCR products were analysed on 3% agarose gels. (DOC) [file pone.0097646.s001.doc]

**Table S1.** Genotype analysis - arrangement and primers.

| Genetic variant | Allele | Sense primer; c_final_ | Antisense primer; c_final_ | Lengh of product, *bp* |
| --- | --- | --- | --- | --- |
| Multiplex 1 |  |  |  |  |
| *ABCG2* c.421C>A | WT | 5´- TCCTTCACCTTTCTTTTCCCCTAG CTTAGA -3´; 250 nmol/L | 5´- TGTTACAAGCCGAAGAGCTGCTG AGAATTG -3´; 250 nmol/L | 393 |
| *SLC2A9* c.844G>A | Mut | 5´- TGGACACTCTAATCCCTGCTGAAA GTCCAT -3´; 250 nmol/L | 5´- AGCTCCAGCACGGACACCAGGCGG ATGCTCCTCTGCAAGT -3´; 250 nmol/L | 154 |
| *SLC2A9* c.881G>A | Mut |  | 5´- AGCCAGGACCTCCTCTACCTCTTG GGATAT -3´; 250 nmol/L | 107 |
| Multiplex 2 |  |  |  |  |
| *ABCG2* c.421C>A | Mut | 5´- TCCTTCACCTTTCTTTTCCCCTAG CTTAGA -3´; 250 nmol/L | 5´- TGTTACAAGCCGAAGAGCTGCTG  AGAATTT -3´; 1000 nmol/L | 393 |
| *SLC2A9* c.844G>A | WT | 5´- TGGACACTCTAATCCCTGCTGAAA GTCCAT -3´; 250 nmol/L | 5´- AGCTCCAGCACGGACACCAGGCGG ATGCTCCTCTGCAAGC -3´; 250 nmol/L | 154 |
| *SLC2A9* c.881G>A | WT |  | 5´- AGCCAGGACCTCCTCTACCTCTTG GGATAC -3´; 500 nmol/L | 107 |

The selected genotypes were determined using multiplex ARMS-PCR. The amplifications were performed in the total volume 10µl using Combi PPP master mix (Top-Bio s.r.o., Prague, Czech Republic) supplied with allele specific primer pairs as it is shown in this table. The cycling conditions were [95/2min;30 x (95/10s, 68/30s); 68/5min] in DNA Engine Dyad PTC-220 (MJ Research, Waltham, Massachusetts). PCR products were analysed on 3% agarose gels.
