# Supplementary material for: Metabolic Syndrome, Alcohol Consumption and Genetic Factors Are Associated with Serum Uric Acid Concentration
Source: PLoS One. 2014 May 14;9(5):e97646. doi: 10.1371/journal.pone.0097646 (PMC4020828; doi:10.1371/journal.pone.0097646)
Supplement: Table S2 — The final multiple linear regression model (Model 1) and the alternative model, with MS predictors replaced by graded MS (Model 2). * log-transformed for the model, coefficient and standard error relevant for the transformed variable. ** values for man/woman because of an interaction with gender present in the model. (DOC) [file pone.0097646.s002.doc]

**Table S2**. The final multiple linear regression model (Model 1) and the alternative model, with MS predictors replaced by graded MS (Model 2).

|  |  | | **Univariate regression model (gender as covariate)** | | | **Multiple regression model 1** | | | **Multiple regression model 2** | | |
| --- | --- | --- | --- | --- | --- | --- | --- | --- | --- | --- | --- |
|  |  | | **Estimate** | **Std. Error** | **p-value** | **Estimate** | **Std. Error** | **p-value** | **Estimate** | **Std. Error** | **p-value** |
| **Conventional predictors** | | |  |  |  |  |  |  |  |  |  |
| Intercept (mutliple regression model) | | |  |  |  | 5,53 | 0,12 | <0.001 | 5,45 | 0,09 | <0.001 |
| Sex; female vs. male | | | -0,353 | 0,019 | < 0.001 | -0,707 | 0,104 | <0.001 | -0,314 | 0,025 | <0.001 |
| Climax (women only); yes/no (%) | | | -0,137 | 0,028 | < 0.001 | 0,096 | 0,026 | <0.001 | 0,122 | 0,027 | <0.001 |
| Age (year) | | | 0,003 | 0,001 | 0,002 | -0.002 | 0.001 | 0.043 | -0,002 | 0,001 | 0,030 |
| Alcohol consumption – beer (0.5L drink/day) | | | -0,027 | -0,013 | 0,045 | - | - | - | 0,035 | 0,012 | 0,003 |
| Alcohol consumption – wine (0.2L drink/day) | | | -0,065 | 0,036 | 0,069 | - | - | - | -0,061 | 0,031 | 0,050 |
| Smoking – smoker; yes/no (%) | | | -0,129 | 0,081 | 0,114 | - | - | - | - | - | - |
| Smoking – former smoker; yes/no (%) | | | -0,073 | 0,075 | 0,329 | - | - | - | - | - | - |
| Smoking – smoked packyears (pack·year) | | | -4*E-9 | 2*E-8 | 0,793 | - | - | - | - | - | - |
| Allopurinol users; yes/no (%) | | | 0,264 | 0,089 | 0,003 | 0,188 | 0,073 | 0,011 | 0,156 | 0,078 | 0,047 |
| Diuretics users; yes/no (%) | | | 0,199 | 0,044 | 0,000 | - | - | - | 0,086 | 0,041 | 0,037 |
| Acetylsalicyl acid users; yes/no (%) | | | -0,001 | 0,058 | 0,991 | - | - | - | - | - | - |
| HA users (women only); yes/no (%) | | | -0,186 | 0,050 | < 0.001 | - | - | - | - | - | - |
| SHT users (women only); yes/no (%) | | | -0,150 | 0,036 | < 0.001 | - | - | - | - | - | - |
| Hormones (women only); yes/no (%) | | | -0,179 | 0,030 | < 0.001 |  |  |  |  |  |  |
| Serum creatinine (µmol/L) | | | 0,004 | 0,001 | < 0.001 | 0,004 | 0,001 | <0.001 | 0,004 | 0,001 | <0.001 |
| **Metabolic syndrome related predictors** | | |  |  |  |  |  |  |  |  |  |
| BMI (kg/m^2^) | | | 0,020 | 0,002 | < 0.001 | 0.001 / 0.017 ** | 0.003 / 0.004** | 0.747 / <0.001** | - | - | - |
| WHR (m/m) | | | 1,082 | 0,166 | < 0.001 | - | - | - | - | - | - |
| Obesity based on MS criteria; yes/no (%) | | | 0,159 | 0,020 | < 0.001 | - | - | - | - | - | - |
| Systolic pressure (mm Hg) | | | 0,002 | 0,001 | 0,000 | - | - | - | - | - | - |
| Diastolic pressure (mm Hg) | | | 0,004 | 0,001 | 0,000 | - | - | - | - | - | - |
| Hypertension; yes/no (%) | | | 0,174 | 0,028 | < 0.001 | 0.075 | 0.025 | 0.003 | - | - | - |
| Hypertension treatment; yes/no (%) | | | 0,207 | 0,031 | < 0.001 | - | - | - | - | - | - |
| Hypertension based on MS criteria; yes/no (%) | | | 0,064 | 0,019 | 0,001 | - | - | - | - | - | - |
| Glycemia (mmol/L) | | | 0,017 | 0,010 | 0,092 | -0,022 | 0,009 | 0,012 | - | - | - |
| Diabetes mellitus (DM); yes/no (%) | | | 0,016 | 0,050 | 0,753 | - | - | - | - | - | - |
| DM treatment; yes/no (%) | | | -0,100 | 0,079 | 0,207 | - | - | - | - | - | - |
| Hyperglycemia based on MS criteria; yes/no (%) | | | 0,116 | 0,024 | < 0.001 | - | - | - | - | - | - |
| Total cholesterol (mmol/L) | | | 0,041 | 0,009 | < 0.001 | - | - | - | - | - | - |
| HDL cholesterol (mmol/L) | | | -0,136 | 0,027 | < 0.001 | - | - | - | - | - | - |
| LDL cholesterol (mmol/L) | | | 0,037 | 0,011 | < 0.001 | - | - | - | - | - | - |
| Reduced HDL-cholesterol based on MS criteria; yes/no (%) | | | 0,138 | 0,027 | < 0.001 | - | - | - | - | - | - |
| Triacylglycerols * (log(mmol/L)) | | | 0,160 | 0,018 | < 0.001 | 0,105 | 0,018 | <0.001 | - | - | - |
| Hypertriacylglycerolemia based on MS criteria; yes/no (%) | | | 0,131 | 0,021 | < 0.001 | - | - | - | - | - | - |
| Hyperlipidemia; yes/no (%) | | | 0,045 | 0,022 | 0,042 | - | - | - | - | - | - |
| GGT * (log(µkat/L)) | | | 0,125 | 0,019 | < 0.001 | 0,064 | 0,017 | <0.001 | - | - | - |
| Number of MS criteria | | | 0,064 | 0,007 | < 0.001 | - | - | - | 0,057 | 0,007 | <0.001 |
| MS; yes/no (%) | | | 0,129 | 0,021 | < 0.001 | - | - | - | - | - | - |
| **Genetic predictors** | | |  |  |  |  |  |  |  |  |  |
| *MTHFR* c.665 C>T | | (TT or CT) vs. CC | 0,021 | 0,020 | 0,291 | - | - | - | - | - | - |
|  | | TT vs. (CT or CC) | 0,063 | 0,032 | 0,051 | - | - | - | - | - | - |
|  | | N of variant alleles | 0,026 | 0,015 | 0,086 | - | - | - | - | - | - |
| *MTHFR* c.1286 A>C | | (CC or AC) vs. AA | -0,017 | 0,019 | 0,388 | - | - | - | - | - | - |
|  | | CC vs. (AA or AC) | -0,072 | 0,032 | 0,025 | -0.064 | 0.026 | 0.014 | -0,055 | 0,028 | 0,049 |
|  | | N of variant alleles | -0,025 | 0,015 | 0,092 | - | - | - | - | - | - |
| *ABCG2* c.421C>A # | | (AA or CA) vs. CC | 0,082 | 0,024 | 0,001 | - | - | - | - | - | - |
|  | | AA vs. (CC or CA) | 0,262 | 0,096 | 0,007 | - | - | - | - | - | - |
|  | | N of variant alleles | 0,084 | 0,022 | < 0.001 | 0.113 / 0.030 ** | 0.026 / 0.037 ** | <0.001 / 0.024 ** | 0,079 | 0,020 | <0.001 |
| *SLC2A9* c.881G>A | | (AA or GA) vs. GG | -0,022 | 0,020 | 0,267 | - | - | - | - | - | - |
|  | | AA vs. (GG or GA) | -0,029 | 0,043 | 0,502 | - | - | - | - | - | - |
|  | | N of variant alleles | -0,019 | 0,016 | 0,247 | -0,044 | 0,014 | 0,001 | -0,044 | 0,015 | 0,003 |
| *SLC2A9* c.844G>A | | (AA or GA) vs. GG | -0,091 | 0,019 | < 0.001 | - | - | - | - | - | - |
|  | | AA vs. (GG or GA) | -0,103 | 0,047 | 0,029 | - | - | - | - | - | - |
|  | | N of variant alleles | -0,078 | 0,016 | < 0.001 | -0,090 | 0,014 | <0.001 | -0,085 | 0,015 | <0.001 |
| **Adjusted R^2^ of the model** | | | | | | R2=0.588 | | | R2=0.531 | | |
| **Adjusted R^2^ of the model without the genetic predictors** | | | | | | R2=0.543 | | | R2=0.491 | | |

* log-transformed for the model, coefficient and standard error relevant for the transformed variable

** values for man / woman because of an interaction with gender present in the model
